# Analgesic Effects of Magnesium Sulphate as an Adjuvant to Fentanyl for Monitored Anaesthesia Care During Hysteroscopy

**DOI:** 10.7759/cureus.43458

**Published:** 2023-08-14

**Authors:** Sonal Mishra, Siddharth Jain, Shobha Purohit, Girdhari Lal, Neelu Sharma

**Affiliations:** 1 Anesthesiology, Sawai Man Singh (SMS) Medical College, Jaipur, IND; 2 Orthopedics, All India Institute of Medical Sciences, Bhopal, IND

**Keywords:** hysteroscopy, opioid-sparing analgesia, opioid-free analgesia, operative hysteroscopy, peri-operative analgesia, magnesium sulphate

## Abstract

Background: Magnesium sulphate (MgSO4) is conventionally used in the treatment of eclampsia, refractive arrhythmias, asthma, etc. In our study, we aimed to study the analgesic effects of MgSO4 as an adjuvant to fentanyl and reduce the intraoperative opioid requirement to decrease their adverse effects.

Methods: A total of 122 patients scheduled for hysteroscopy were randomly divided into two groups. Patients in the magnesium group (group A) received intravenous MgSO4 50 mg/kg in 100 ml of isotonic saline over 15 minutes before anaesthesia induction and then 15 mg/kg per hour by continuous intravenous infusion. Patients in the control group (group B) received an equal volume of isotonic saline as a placebo. All the patients were induced with fentanyl and propofol. Perioperative haemodynamic monitoring and postoperative assessment of pain were done.

Results: Only 18% of the patients in group A required rescue analgesics as compared to 39.3% of patients in group B. The patients receiving MgSO4 displayed lower verbal numeric rating scale scores in the postoperative period. In addition, the intraoperative requirement of fentanyl (101 (21.33) vs. 144 (28.4) µg, mean (SD)) and propofol (121 (13.3) vs. 140 (16.5) mg, mean (SD)) was significantly lower in group A as compared to that in group B.

Conclusion: MgSO4, when administered as an adjuvant to opioids, provided effective postoperative analgesia thereby reducing the need for rescue analgesics. It also decreases intraoperative fentanyl consumption and its dose-related side effects.

## Introduction

Minimally invasive endoscopic procedures, such as hysteroscopy, are frequently carried out in gynaecological hospitals as a daycare procedure. The distension of the uterus and the handling of the instruments might cause pain and discomfort to the patient. Several analgesic agents, including opioids, non-steroidal anti-inflammatory drugs (NSAIDs), and local anaesthetics are commonly used agents to reduce the pain caused by hysteroscopy. Opioids are the most commonly used agents for managing pain but the dose-related side effects like respiratory depression and postoperative nausea and vomiting (PONV) limit their use [1]. Therefore, there is a need for alternative analgesic agents with fewer side effects.

Magnesium sulphate (MgSO4) has been suggested as a potential analgesic agent in various surgical procedures like abdominal hysterectomy, hysteroscopy, arthroscopic knee surgery, inguinal hernia surgery cholecystectomy, and even spine surgeries [2-8]. It has also been found to reduce the consumption of anaesthetic agents, blunt the sympathetic response to laryngoscopy and intubation, and enhance the effect of neuromuscular blockers. In addition to the treatment of magnesium deficiency, MgSO4 is used in the treatment of eclampsia, torsades de pointes, status asthmaticus, etc.

With this background, this clinical study was conducted to study whether MgSO4 has good analgesic effects as an adjuvant in patients undergoing hysteroscopy. The primary objective was to study the proportion of patients in need of rescue analgesia within four hours postoperatively, and the secondary objectives were to compare the haemodynamic variables between the groups and the side effects of receiving MgSO4 and placebo.

## Materials and methods

A double-blinded, randomized control trial was conducted in a tertiary-level healthcare hospital in India from 2021 to 2022 on 122 females who underwent hysteroscopy after taking due permission from Sawai Man Singh (SMS) Medical College Ethics Committee (1159/MC/EC/2021). This trial was registered under the Clinical Trials Registry of India. A sample of 61 cases in each group was calculated at 95% confidence and 80% power to predict the expected difference of 23% in the proportion of cases who need rescue analgesia between two different groups receiving intravenous magnesium sulphate and an equal volume of isotonic saline as a placebo.

Inclusion criteria

Females aged 18-55 years old undergoing hysteroscopy with American Society of Anesthesiologists (ASA) grade I and II who were willing to provide informed consent were included in this study.

Exclusion criteria

Patients with ASA grade III and IV who had either a history of drug allergy, pre-existing renal or hepatic dysfunction, pre-existing cardiovascular disease, or BMI ≥ 30 kg/m2 (difficult airway) were excluded.

Study procedure

The patients were randomly divided into two groups (sealed envelope method) to receive MgSO4 or a placebo. The participating clinicians were given randomly generated treatment allocations within sealed opaque envelopes. Once a patient had consented to enter a trial, an envelope was opened and the patient was then offered the allocated treatment regimen. An anaesthetist who did not participate in the study prepared the drug infusion according to the group assigned. Anaesthesia providers, patients, and all investigators were blinded to group assignment until the completion of the study.

The MgSO4 group (group A) received 50 mg/kg MgSO4 in 100 ml of isotonic saline over 15 minutes whereas the control group (group B) received an equal volume of isotonic saline. All patients underwent extensive pre-anaesthesia examinations. Upon reaching the operating theatre (OT), their fasting status, pre-anaesthetic checkup (PAC), and consent were verified. Baseline haemodynamic parameters like heart rate (HR), systolic blood pressure (SBP), diastolic blood pressure (DBP), mean blood pressure (MBP), and oxygen saturation (SPO2) were measured. The patients in group A received the MgSO4 infusion, while group B patients received an equal volume of saline before induction of anaesthesia. All patients received a 1.5 µg/kg intravenous bolus dose of fentanyl, 0.1 mg/kg of midazolam, and 200 µg of glycopyrrolate four minutes prior to the surgery. Propofol 1.5 mg/kg was used to start the sedation process; it was subsequently continued at a rate of 4-6 mg/kg per hour. The propofol and study medication infusions were stopped as soon as the gynaecologists declared the procedure to be over. During the surgery, 100% oxygen was delivered to all patients via a face mask for spontaneous breathing. Patients were managed with jaw thrust if SpO2 was less than 95%, and assisted ventilation was used to manage SpO2 below 90%. Mephentermine (6 mg IV stat) was administered to manage episodes of hypotension (SBP ≤ 90 mmHg). After the procedure, the patients were shifted to the recovery room. Intraoperative and postoperative haemodynamic vitals were recorded at various time intervals. The total dosage of anaesthetic drugs (propofol and fentanyl) consumed, the time it took for patients to regain consciousness, postoperative pain scores calculated using the verbal numeric rating scale (VNRS; 0 = no pain, 4-6 = moderate pain, 7-9 = severe pain, and 10 = worst pain), the total amount of rescue analgesic used, instances of PONV, and other side effects were all noted. If VNRS ≥ 4, the patients got diclofenac 75 mg (IV stat) as a rescue analgesic, and episodes of PONV were treated with ondansetron 4 mg/kg intravenously.

Data analysis

Data collected were compiled in a Microsoft Excel spreadsheet (Microsoft Corporation, Redmond, WA) as a master chart. Data were presented as tables, figures, and charts. Nominal/categorical variables were summarized as frequency and percentage and were analysed using the chi-square test. Continuous variables were summarized as mean and standard deviation and were analysed using the independent sample t-test for comparison between the two groups. The ordinal variable (VNRS score) was expressed as median and IQR and was analysed using the Mann-Whitney U test for comparison between the two groups. A p-value ≤ 0.05 was taken as statistically significant. All statistical analyses were done using Epi Info version 7.2.1.0 statistical software (CDC, Atlanta, Georgia).

## Results

The patient characteristics in both groups are described below (Tables 1, 2). The groups were similar with respect to age, weight, and the American Society of Anesthesiologists (ASA) grade.

**Table 1:** Demographic profile of patients in both study groups

	Group A	Group B	P-value
Age, years	38.02 (7.52)	37.72 (8.06)	0.12
Weight, kg	57.20 (7.92)	56.23 (9.08)	0.532

**Table 2:** Distribution of patients according to the American Society of Anesthesiologists (ASA) grade Chi-square = 0.534 with one degree of freedom; P = 0.465.

ASA grade	Group A		Group B		Total	
	N	%	N	%	N	%
I	24	39.3	29	47.5	53	43.4
II	37	60.7	32	52.5	69	56.6
Total	61	100	61	100	122	100

The mean baseline heart rate was 85.05 ± 12.22 bpm in group A and 86.77 ± 9.63 bpm in group B and was found to be statistically insignificant (p = 0.389). Likewise, the average baseline mean arterial pressure (MAP) of groups A and B was 93.07 ± 6.1 mmHg and 92.07 ± 5.26 mmHg, respectively, and was found to be statistically insignificant (p = 0.334).

The difference in mean heart rate at one, five, 10, and 15 minutes after induction of anaesthesia between group A and group B was 75.34 ± 12.27/minute, 72.41 ± 12.42/minute, 73.13 ± 14.69/minute, and 73.05 ± 10.65/minute and 82.77 ± 9.86/minute, 80.92 ± 11.4/minute, 80.46 ± 13.56/minute, and 84.59 ± 11.75/minute, respectively, which was found to be statistically significant and it remained statistically significant throughout the procedure (p < 0.001).

The mean baseline SBP was 119.64 ± 7.89 mmHg in group A and 118.41 ± 6.83 mmHg in group B and was found to be statistically insignificant (p = 0.359). Likewise, the average baseline mean DBP of groups A and B was 79.79 ± 5.22 mmHg and 78.9 ± 4.48 mmHg, respectively, and was found to be statistically insignificant (p = 0.317).

The MAP at one minute, five minutes, 10 minutes, and 15 minutes after induction was found to be statistically significant (p < 0.001), whereas it was found to be statistically insignificant throughout the rest of the procedure.

In group A, the postoperative heart rate at one minute was 78.87 ± 15.85/minute, and at 15 minutes, 30 minutes, one hour, and four hours, the heart rate was 78.15 ± 15.45/minute, 77.28 ± 14.41/minute, 75.08 ± 14.45/minute, and 75.08 ± 14.45/minute, respectively. In group B, the postoperative heart rate at one minute was 79.79 ± 22.02/minute, and at 15 minutes, 30 minutes, one hour, and four hours, the heart rate was 79.11 ± 20.31/minute, 77.08 ± 21.28 /minute, 79.77 ± 19.47/minute, and 79.95 ± 20.09/minute, respectively. The difference in heart rate in the postoperative period between the study groups was found to be statistically insignificant. In group A, the postoperative SBP at one minute was 105.57 ± 10.92 mmHg, and at 15 minutes, 30 minutes, one hour, and four hours, the SBP was 107 ± 11.64 mmHg, 109 ± 11.85 mmHg, 109.95 ± 8.89 mmHg, and 110.56 ± 9.33 mmHg, respectively. In group B, the postoperative SBP at one minute was 104.15 ± 10.25 mmHg, and at 15 minutes, 30 minutes, one hour, and four hours, the SBP was 104.21 ± 10.66 mmHg, 107.66 ± 10.45 mmHg, 108.07 ± 9.48 mmHg, and 108.56 ± 7.35 mmHg, respectively. The difference in SBP in the postoperative period between the study groups was found to be statistically insignificant. In group A, the postoperative DBP at one minute was 70.41 ± 7.3 mmHg, and at 15 minutes, 30 minutes, one hour, and four hours, the DBP was 71.41 ± 7.74 mmHg, 72.67 ± 7.94 mmHg, 73.28 ± 5.96 mmHg, and 73.72 ± 6.21 mmHg, respectively. In group B, the postoperative DBP at one minute was 69.49 ± 6.81 mmHg, and at 15 minutes, 30 minutes, one hour, and four hours, the DBP was 69.51 ± 7.12 mmHg, 70.8 ± 7.01 mmHg, 72.08 ± 6.3 mmHg, and 72.33 ± 4.89 mmHg, respectively. The difference in DBP in the postoperative period between the study groups was found to be statistically insignificant. In group A, the postoperative MAP at one minute was 82.13 ± 8.47 mmHg, and at 15 minutes, 30 minutes, one hour, and four hours, the MAP was 83.28 ± 9.03 mmHg, 84.74 ± 9.21 mmHg, 85.56 ± 6.93 mmHg, and 85.98 ± 7.22 mmHg, respectively. In group B, the postoperative MAP at one minute was 80.98 ± 7.97 mmHg, and at 15 minutes, 30 minutes, one hour, and four hours, the MAP was 81.07 ± 8.26 mmHg, 82.39 ± 8.11 mmHg, 84.07 ± 7.37 mmHg, and 84.38 ± 5.69 mmHg, respectively. The difference in MAP in the postoperative period between the study groups was found to be statistically insignificant.

Total intraoperative consumption of propofol and fentanyl is demonstrated in Table 3. The patients in the control group consumed a significantly higher amount of propofol and fentanyl.

**Table 3:** Total consumption of fentanyl and propofol

	Group A	Group B	P-value
Propofol (mg)	121.5 (13.3)	140.7 (16.5)	<0.001
Fentanyl (mcg)	101 (21.33)	144 (28.4)	<0.001

Postoperatively, all the patients were haemodynamically stable. None of the patients demonstrated bradycardia (heart rate < 60/minute), hypotension (MAP 20% lower than baseline), or respiratory depression during the postoperative monitoring period in the recovery room.

The groups that received MgSO4 had better postoperative pain control, which was evident with significantly lower VNRS scores (p < 0.001) (Table 4).

**Table 4:** Comparison of postoperative VNRS scores between the study groups VNRS: verbal numeric rating scale.

Time	Group A, median (range)	Group B, median (range)	P-value
1 minute	1 (0, 4)	2 (0, 4)	0.026 (significant)
15 minutes	2 (1, 4)	3 (1, 6)	<0.001 (significant)
30 minutes	2 (1, 5)	3 (2, 6)	0.009 (significant)
1 hour	1 (0, 5)	2 (1, 5)	0.029 (significant)
4 hours	1 (0, 3)	2 (1, 4)	0.029 (significant)

Thus, the total number of patients that were in need of rescue analgesia was lesser in group A (Table 5).

**Table 5:** Distribution of patients according to the need for rescue analgesia Chi-square = 5.769 with one degree of freedom; p = 0.016 (S).

Rescue analgesia	Group A		Group B		Total	
	N	%	N	%	N	%
Yes	11	18	24	39.3	35	28.7
No	50	82	37	60.7	87	71.3
Total	61	100	61	100	122	100

In group A, 10 patients had hypotensive episodes and five patients experienced PONV. Whereas, 14 patients in group B had intraoperative hypotension and eight subjects experienced PONV. The difference between the study groups was found to be statistically insignificant.

## Discussion

Hysteroscopy is performed as a daycare procedure nowadays, allowing for shorter hospital stays, early mobilization, and lower expenses. Postoperative analgesia is always underestimated because it is thought to be a less invasive technique. Effective pain management is essential for the comfort of the patient. Uterine and cervical dilatation and intrauterine tissue extraction both result in a considerable amount of pain, which requires effective analgesia. Usually, opioids are used during surgery to effectively manage pain [1]. But their side effects such as respiratory depression and PONV limit their use. The concept of multimodal analgesia techniques works with the aim of improving analgesia by combining two or more analgesic agents and or techniques and reducing opioid consumption and minimizing opioid-related adverse effects.

Multimodal analgesia is a pharmacologic method of pain management that combines various groups of drugs for pain relief. For instance, opioids can be combined with non-opioids according to the individual situation. A local anaesthetic block can also be combined with a systemic analgesic for the desired results. The most commonly combined medication groups include local anaesthetics, opioids, NSAIDs, acetaminophen, and alpha-2 agonists. Using multiple analgesic drugs, with different modes of action, simultaneously, form the key underlying principle of the concept of multimodal analgesia. It aims at providing adequate postoperative analgesia and minimizing opioid-related side effects. This study demonstrates that MgSO4 administration provides effective postoperative analgesia and reduces the need for rescue analgesia with a significant reduction in intraoperative fentanyl and propofol consumption, and thus can be successfully used as an adjuvant to fentanyl.

MgSO4 acts as an antagonist at N-methyl-D-aspartate (NMDA) receptors to exert its antinociceptive effects. As a result, the excitatory neurotransmitters (such aspartate and glutamate) released from the peripheral nociceptive stimuli are prevented from activating NMDA receptors in the dorsal horn of the spinal cord [9,10]. Sweating, elevated blood pressure, and tachycardia are all autonomic reactions that are used empirically to assess the adequacy of analgesia during general anaesthesia. They have little predictive specificity, are easily obscured by drugs (such as beta-blockers), and exhibit interpersonal variability. The MgSO4 group's much-reduced heart rates and blood pressure readings may have been the result of adequate analgesia. The patients in the magnesium group experienced little to no pain upon recovery, which was clearly evident by the postoperative VNRS scores. In our study, group A had considerably lower VNRS ratings than group B, and fewer patients in this group needed rescue analgesics. Extensive research has been done to study the analgesic effects of MgSO4 wherein it was successfully concluded that the patients who received a perioperative infusion of MgSO4 in surgeries under general anaesthesia experienced less postoperative pain, which was evident by pain scores (visual analogue scale, numeric rating scale, and VNRS) that were recorded [7,10-14].

The modulation of the effects of anaesthesia may be due to the competitive blockade of calcium by magnesium at presynaptic calcium channels, as established by Sasaki and colleagues [15]. Various studies have shown that preemptive administration of MgSO4 significantly reduced the consumption of propofol [4,12,16-18], whereas Schulz-Stubner found that propofol consumption remained constant irrespective of MgSO4 infusion (Table 6) [19]. Propofol's anaesthetic, hypnotic, and anticonvulsant effects could be attributed to NMDA-mediated excitatory neuronal transmission. MgSO4, a non-competitive NMDA blocker, might therefore enhance the anaesthetic effects of propofol via this method. Due to its role as an NMDA receptor antagonist and its decreasing effect on the release of neurotransmitters from presynaptic terminals, magnesium oxide has a depressant effect on the central nervous system (CNS). The low dosage of fentanyl consumed in the magnesium group suggests effective intraoperative analgesia. Koinig et al. [3] demonstrated that in patients with similar levels of surgical stimulation, MgSO4 can be successfully used as an adjuvant to perioperative analgesia by reducing the fentanyl requirements. A systematic review conducted by Rodríguez-Rubio et al. analysed 20 clinical trials for qualitative data and 19 for quantitative data. They found that using MgSO4 through an IV route before surgery reduces the amount of anaesthesia needed for both starting (induction) and maintaining (maintenance) anaesthesia. It also reduces the need for neuromuscular non-depolarizing blocking agents and the use of fentanyl during surgery [20].

**Table 6:** Past studies related to the consumption of propofol where magnesium sulphate was used as a rescue analgesic.

S. No.	Authors	Year of publishing	Propofol consumption
1.	Schulz-Stubner et al. [19]	2001	Unchanged
2.	Telci et al. [4]	2002	Reduced
3.	Altan et al. [17]	2005	Reduced
4.	Seyhan et al. [18]	2006	Reduced
5.	Kiran et al. [12]	2011	Reduced
6.	Cizmeci et al. [16]	2007	Reduced

The lower heart rates may also be attributed to the fact that MgSO4 is known to have a negative ionotropic action and cause myocardial depression and cardiac arrest in massive doses. This decrease in heart rate may be brought about by both indirect and direct inhibitory effects of magnesium (Mg) on the sinoatrial (SA) node. As for the indirect effect, Stanbury [21] found that Mg produces a short-lived blockade of sympathetic ganglia and prevents the stimulating effect of K+ and Ach on the superior cervical ganglion while, for the direct inhibitory effect. Opthof et al. [22] demonstrated that Mg itself decreases the depolarization of pacemaker cells of the SA node. Herroeder et al. [23] demonstrated the inhibitory effects of Mg on the release of catecholamines. Reinhart et al. have demonstrated that MgSO4 has a direct effect on blood vessels by inhibiting calcium, acetylcholine, angiotensin, and epinephrine, which causes a fall in blood pressure [24]. Thus, magnesium has been shown to vasodilate blood vessels in many vascular beds (mesenteric, skeletal muscle, uterine, cerebral, coronary, and the aorta), which shows its direct correlation in decreasing blood pressure. Inhibition of calcium channels and decreased activation of Ca-ATPase and Na-K ATPase is responsible for the fall in blood pressure seen with MgSO4. Moreover, Mg can induce the production and secretion of prostacyclin (PGI2) and reduce the activity of the angiotensin-converting enzyme.

Although MgSO4 is particularly safe when administered in therapeutic dosage, some undue side effects were encountered in our study. Findings have shown that during the initiation of IV MgSO4 infusion, especially due to the rapid administration of the initial bolus dose, a feeling of warmth may occur. Although in the current study, IV MgSO4 was given over 15 minutes with slow infusion, in our study, 15 patients of group A complained of a burning sensation during the administration of bolus dose. However, because anaesthesia was administered immediately after this effect, these hot flushes lasted only a few minutes. This was consistent with the study conducted by Cizmeci et al. [16] where the patients complained of a burning sensation during the administration of a bolus dose. No episodes of bradycardia were reported in either group. Episodes of hypotension occurred in 10 patients in group A whereas 14 patients had hypotension in group B, which were managed by fluid boluses and injection of mephentermine (6 mg single dose). PONV occurred in five patients in group A and in eight patients in group B, which were managed by ondansetron 4 mg injection.

In our study, some limitations should be noted. We did not measure the preoperative and postoperative serum magnesium levels; also for the awareness during anaesthesia, bispectral index (BIS) monitoring was not done, which could have provided more information. A patient’s perception of pain can be influenced by a variety of factors such as personality and psychological factors, which could affect the results of the study.

## Conclusions

In comparison to fentanyl alone, MgSO4 when used as an adjuvant is more helpful in lowering postoperative pain and the need for rescue analgesics in patients having hysteroscopy. Also, it offers a comfortable and smooth recovery of consciousness and stable haemodynamic characteristics. Hence, MgSO4 can be utilized safely as an adjuvant to total intravenous anaesthesia in day surgery procedures like hysteroscopy.
